# Societal recovery trajectories in people with a psychotic disorder in long term care: a latent class growth analysis

**DOI:** 10.1007/s00127-024-02715-0

**Published:** 2024-07-30

**Authors:** Stijn Crutzen, Simone R. Burger, Ellen Visser, Helga K. Ising, PHAMOUS investigators, Johan Arends, Frederike Jörg, Gerdina Hendrika Maria Pijnenborg, Wim Veling, Mark van der Gaag, Stynke Castelein

**Affiliations:** 1 Lentis Psychiatric Institute, Lentis Research, Groningen, The Netherlands; 2 Department of Clinical Psychology, VU University and Amsterdam Public Health Research Institute, Amsterdam, The Netherlands; 3 Department of Psychosis Research and Innovation, Parnassia Psychiatric Institute, The Hague, The Netherlands; 4 University Medical Center Groningen, Rob Giel Research Center, University of Groningen, University Center Psychiatry, Groningen, The Netherlands; 5 Rivierduinen Institute for Mental Health Care, Leiden, The Netherlands; 6 Faculty of Behavioural and Social Sciences, University of Groningen, Grote Kruisstraat 2/1, Groningen, 9712 TS The Netherlands

**Keywords:** Psychotic disorder, Societal recovery, Latent class growth analysis, Long term care

## Abstract

**Purpose:**

For many individuals with a psychotic disorder societal recovery is not accomplished. Research on societal recovery trajectories is mostly focussed on patients with a first episode psychosis. The present study aims to identify distinct societal trajectories in those with long duration of illness, through the identification of patient subgroups that are characterized by homogeneous trajectories.

**Methods:**

Longitudinal data were used from an ongoing dynamic cohort in which people with a psychotic disorder receive yearly measurements to perform a latent class growth analysis. Societal functioning was assessed with the Functional Recovery tool, consisting of three items (1) daily living and self-care, (2) work, study and housekeeping, and (3) social contacts. Furthermore, logistic regression was used to compare subgroups with similar societal recovery at baseline, but distinct trajectories.

**Results:**

A total of 1476 people were included with a mean treatment time of 19 years (SD 10.1). Five trajectories of functioning were identified, a high stable (24.5%), a medium stable (28.3%), a low stable (12.7%), a high declining (11.2%) and a medium increasing subgroup (23.3%). Predictors for not deteriorating included happiness, recent hospitalisation, being physically active, middle or higher education and fewer negative symptoms. Predictors for improving included fewer positive and negative symptoms, fewer behavioural problems and fewer physical and cognitive impairments.

**Conclusion:**

While the majority of individuals show a stable trajectory over four years, there were more patients achieving societal recovery than patients deteriorating. Predictors for improvement are mainly related to symptoms and behavioural problems, while predictors for deteriorating are related to non-symptomatic aspects such as physical activity, happiness and level of education.

**Supplementary Information:**

The online version contains supplementary material available at 10.1007/s00127-024-02715-0.

## Introduction

A first episode of psychosis (FEP) can have major impact on various aspects of life. In most cases it is very disruptive for someone’s education, independence, and ability to develop and maintain relationships and employment [1]. Problems in these areas often precedes the FEP due to the cognitive deficits already present during the prodromal phase of psychosis [2]. People with a psychotic disorder consider societal recovery (an individual’s ability to maintain interpersonal relationships, work or study, and live independently [3]) among their highest treatment priorities [4, 5]. While a majority accomplishes clinical recovery after a FEP [6], most patients are not able to reach full societal recovery [7]. Only about one-third of the patients is employed after eight years following a FEP [7]. Moreover, after a mean follow up of about ten years only about 20% has a relationship. This is in part due to the cognitive problems that often persist regardless of clinical recovery [2].

Recovery of a psychotic disorder is characterized by heterogeneity [8]. The trajectories of clinical and societal recovery can vary greatly [9, 10]. For both clinical and societal recovery the first years after a FEP are considered essential [11–13]. Because of this early detection and early treatment have been prioritized, in part to facilitate societal recovery and to minimize cognitive decline [2]. The importance of early treatment is further illustrated by finding that the duration of untreated psychosis and cognitive impairment are important predictors for societal recovery after a FEP [14]. Recent studies have focused on identifying distinct recovery trajectories during the crucial first years after a FEP [15–18]. When looking at societal recovery, two recent studies have identified distinct societal recovery trajectories during the first year after a FEP [15, 16]. Van der Ven et al. (2020) analysed two components of societal functioning namely social and occupational functioning during the first year after a FEP. They distinguished the following four trajectories. In one of the trajectories, the majority (58%) displayed moderate social functioning and low yet increasing occupational functioning [16]. Next, a minority (9%) showed low functioning with only modest improvements, 18% maintained a moderate level and 15% showed a stable trajectory of high functioning. Hodgekins et al. (2015) analysed social functioning trajectories which was measured with the time use survey, which measure time spend in several activities (work, education, voluntary work, housework and childcare, leisure, and sports). In contrast, they identified three trajectories, a low stable (66%), a moderate increasing (27%) and a high decreasing (7%) social recovery trajectories in the first year after FEP [15].

Still, about 80% of patients experience at least one relapse within five years after a FEP and the majority is not able to reach both clinical and societal recovery [11, 19]. While research examining trajectories of societal functioning after a FEP during this crucial phase of recovery are essential, little is known about the trajectories of patients that transition to long term care. This distinct group of patients did not manage to fully recover during the critical treatment period in the first years after their FEP [11–13].This gap in the literature results in a challenge for both patients and clinicians: it limits insight in future perspectives for patients. The importance of providing insight in future expectations through psychoeducation for people with psychosis and their close-ones has been demonstrated multiple times [20].

The present study aims to identify distinct trajectories, through the identification of subgroups that are characterized by similar trajectories of societal recovery over time in people with a psychotic disorder. Specifically, patients with an illness duration of five years or more will be followed. Additionally, baseline differences between the identified subgroups will be studied, to investigate the characteristics of distinct societal recovery trajectories.

## Methods

### Design & participants

Longitudinal data from the Pharmacotherapy Monitoring and Outcome Survey (PHAMOUS) were analysed. This was done with the primary goal of identifying subgroups with distinct growth trajectories of social recovery. The secondary goal was to identify predictors for subgroup membership. PHAMOUS is an ongoing dynamic cohort in which patients with a psychotic spectrum disorder receive yearly measurements for as long as they are in care of one of the four participating mental health institutions in the Northern part of the Netherlands [21]. PHAMOUS was designed as Routine Outcome Monitoring (ROM) in order to evaluate treatment on both a patient level and a population level. This cohort has been described in more detail elsewhere [21]. Patients were included when they met the criteria for diagnoses of schizophrenia, schizoaffective disorder and other psychotic disorders according to the DSM-IV/DSM-V [22, 23] and were at least 18 years old. Participants were included when they had at least two societal recovery measurements during a four-year period within the observation period of 2012 to 2019, e.g., someone with a measurement in 2012 and 2017 was not included, but someone with a measurement in 2016 and 2018 was included. The first measurement used needed to be at least five years after their FEP. So, all baseline measurements used were at least five year after the FEP. Data were used from a pre-COVID 19 pandemic period to eliminate the influence of the pandemic on societal recovery.

### Ethics

This study was performed in line with the principles of the Declaration of Helsinki. Ethical approval was granted by the Medical Ethics Review Board of the University Medical Center of Groningen in the Netherlands (approval number METc 2015/347). Formal informed consent was not required since the PHAMOUS screening is part of usual care, as such participants had the option to opt out from the use of their pseudonymised data for scientific purposes.

### Outcomes

#### Societal recovery

The main outcome was societal recovery as assessed with the Functional Recovery tool (FRt) [24, 25].Societal recovery was scored by a clinician on three domains; 1) daily living and self-care, 2) work, study and housekeeping, and 3) social contacts. Each domain contained one item which was scored on range from ‘independent’ (score 0), ‘partially independent’ (score 1) and ‘not independent’ (score 2). A score of zero is indicative of normal functioning, which is defined as an absence of any problem or need for help in this domain. A score of one represents a clear lack of proficiency in this domain for which support is received. Finally, a score of two represents a complete inability to function according to the norm in this domain. For the analyses the reversed sum score of the FRt was used in order to make the interpretation more intuitive. By using the reversed score, a higher sum score on the FRt represent better functioning. A sum score of five or six is indicative of societal recovery.

#### Predictors

In a secondary analysis, multiple logistic regression analyses were performed to identify predictors for class membership of distinct growth trajectories. Based on literature, variables were selected from the list of available variables in the PHAMOUS database which had no more than 30% missing [14–18, 26–30]. Not only predictors for societal recovery in patients with a long duration of illness identified in the literature were included, but also predictors of societal recovery in patients after a FEP. Predictors only needed to be mentioned once in literature in order to be included. Predictors from FEP literature and predictors that are only mentioned once are included because relatively little is known about predictors for societal recovery in patient that are in long term care. Therefore, an exploratory pragmatic approach was used prioritizing not missing potential predictors. Predictors were extracted from the first measurement in which a patient was included in the LCGA. Below all the included predictors are described in detail. How certain predictors are defined is largely based on how they were measured and included in the PHAMOUS dataset. All the included predictors are listed in Tables 1 and 2 of the appendix 1.

#### Demographics

Age, sex and highest level of education were used as potential predictors. Education was categorized in lower (no education, primary school or prevocational education), middle (vocational education) and higher education (senior general secondary education, pre-university education, higher vocational education and university). The level of education was added to the PHAMOUS screening in 2014, so when the baseline year was 2012 or 2013 the educational level registered in 2014 or later screening years was used.

#### Diagnosis and comorbidities

The main psychotic disorder diagnosis at baseline or the most recent available diagnosis before baseline based on the DSM-5 classification was used. Participants for whom no main disorder classification was available at or before baseline were classified as unknown disorder [22]. Additionally, age at onset and time since onset were used as potential predictors. Substance use was captured in four binary predictors, namely alcohol use (yes/no), cannabis use (yes/no), nicotine use (yes/no) and other drug use (yes/no). The presence of one or more major somatic comorbidities was used as a binary predictor (diabetes, cardiovascular disease, hyperchloremia, osteoporosis, thyroid disease and/or epilepsy).

#### Symptoms and functioning

The Positive and Negative Syndrome Scale (PANSS) was used to measure the severity of psychotic symptoms [31]. The PANSS consists of 30 items that represent different psychiatric symptoms and are rated by a trained clinician assessing a semi-structured interview with the participant. The items are sorted in three subscales: positive symptoms (P1-P7), negative symptoms (N1-N7) and general symptoms (G1-G16). Each item is scored on a 7-point Likert-scale ranging from 1 (absence of symptoms) to 7 (extreme severity). A total score was calculated for every subscale, which were used as predictors. The Health of the Nation Outcome Scales (HoNOS) was used to measure psychosocial functioning [32]. The HoNOS is a 12-item instrument containing four subscales: behavioural problems (item 1–3), cognitive and physical limitations (item 4–5), symptomatology (item 6–8), and social problems (item 9–12). The social problem subscale was not used due to overlap with the outcome measure and the symptomatology subscale was not used due to overlap with the PANSS. The HoNOS is rated by a clinician using a 5-point Likert scale where a score of 0 indicates no problem and a score of 4 indicates a very severe problem. Whether or not a participant was hospitalised in the last year due to relapse was used as a predictor. The Single-Item Happiness Question (SIQ) (scale: 0–10) was used as a measure for happiness, it was dichotomised with a cut-off value of seven [33]. Physical activity was included as a binary variable where participants were considered physically active when they reported that they exercised moderately intense for 30 min for at least four times per week.

#### Medication

The type of antipsychotic medication was categorised in a strong dopamine antagonist (Haloperidol, Risperidone, Olanzapine, Zuclopenthixol and Perphenazine), a partial dopamine antagonist (Aripiprazole, Ziprasidone and Amisulpride) or a weak dopamine antagonist (Clozapine, Quetiapine and Sulpiride) and the total haloperidol equivalent dose was calculated based on the defined daily dose [34]. Whether or not antipsychotic medication was used was not included because the vast majority (approximately 95%) used at least one antipsychotic.

### Analyses

#### Latent class growth analysis

To identify subgroups with distinct growth trajectories of societal recovery over time, Latent Class Growth Analysis (LCGA) was performed in Mplus version 7 for Mac [35, 36]. LCGA is a type of growth mixture modelling that identifies homogeneous subgroups (latent classes) with similar growth over time based on one outcome variable (societal functioning). The number of latent classes were increased until the best-fitting model was established to identify the optimal number of classes [35]. A range of fit indices were used to identify the model that fitted best, while taking interpretability into account. The Bayesian Information Criterion (BIC) is an index based on the log-likelihood of the model and the number of model parameters. The smallest BIC suggests the best model fit. Also, the Vuong-Lo-Mendell-Rubin Likelihood Ratio Test [37] and the Bootstrapped Likelihood Ratio Test (BLRT) were used. A significant LMR-LRT or BLRT value indicates that a K-class model fits the data better than a model with K-1 classes. Because mixture models are susceptible to converge on local rather than global solutions [38], multiple random starting values were used for the estimated models (10 repeats with 2 final optimizations). A full-information maximum likelihood algorithm was used to deal with missing societal recovery data.

#### Logistic regression

Logistic regression with a backward elimination approach was used to compare subgroups with similar societal recovery at baseline, but distinct trajectories identified in the LCGA. Variables were included in the logistic regression analyses when in univariate analyses a P-value ≤ 0.1 was found (a relatively high P-value was used to ensure no potentially relevant predictors were discarded in this phase; t-tests or Mann-Whitney tests for continuous variables and Chi-square tests for categorical variables). The variance inflation factors (VIFs) were calculated to assess multicollinearity. Multiple chained imputations were used to deal with missing data. In total 20 imputed datasets were created. For continuous variables predicted mean matching (nearest neighbours = 10) was used and for binary variables logistic regression was used to impute the data. Age, gender and LCGA-class identity were used as independent variables for the imputation. Categorical variables were not imputed, instead the missing category was used as one of the categories in the regression analyses.

## Results

Only patients with at least two measurements were included, 40.0% had three FRt measurement and 22.7% had four measurements. In the first year after their baseline measure 73.3% had a FRt measurement, 63.7 had a FRt measurement in the second year and 48.4% had a measurement in their third year after their baseline measurement. A total of 1476 patients were included in the LCGA. Table 1 shows the baseline characteristics. On inspection the patients that fulfilled the inclusion criteria for the LCGA analysis were similar in age, sex and the percentage of patients that are in symptomatic remission compared to the overall PHAMOUS cohort [21]. On average they were 45 years old, about two-third was male, on average they received mental health care for 19.2 years, about a quarter was in societal recovery and about half was in symptomatic remission at baseline (first measurement used in the LCGA analysis).

**Table 1:** Characteristics of patients included in the LCGA of the first year that was included in the LCGA

Variables		Mean (SD) or %
Age in years, mean (SD)		44.5 (10.7)
Sex (male), %		66.8%
Age at onset in years, mean (SD)		25.4 (9.1)
Years receiving care, mean (SD)		19.2 (10.1)
Non-white ethnicity, %		11.9%
Antipsychotic use, %		95.3%
Mean PANSS general psychopathology scale, mean (SD)		1.54 (0.75)
Mean PANSS positive symptoms scale, mean (SD)		1.96 (0.98)
Mean PANSS negative symptoms scale, mean (SD)		2.13 (1.08)
Symptomatic remission ^a^, %		48.3%
Societal functioning ^b^, mean (SD)		3.29 (1.74)
	Low societal functioning (0–2), %	34.4%
	Medium societal functioning (3–4), %	38.7%
	High societal functioning (5–6), %	27.0%
Main diagnosis, %		
	Schizophrenia	49.7%
	Delusional disorder	1.5%
	Schizophreniform disorder	5.3%
	Substance abuse psychosis	7.9%
	Schizoaffective disorder	13.1%
	Psychosis NOS	2.8%
	Unknown disorder	19.7%
Education, %		
	Lower education	45.7%
	Middle education	25.9%
	Higher education	28.5%

^a^ A score of three or less on the eight PANSS-remission items over a period of at least 6 months [39]

^b^ Scored on a scale from 0–6

### LCGA

**Fig. 1:**
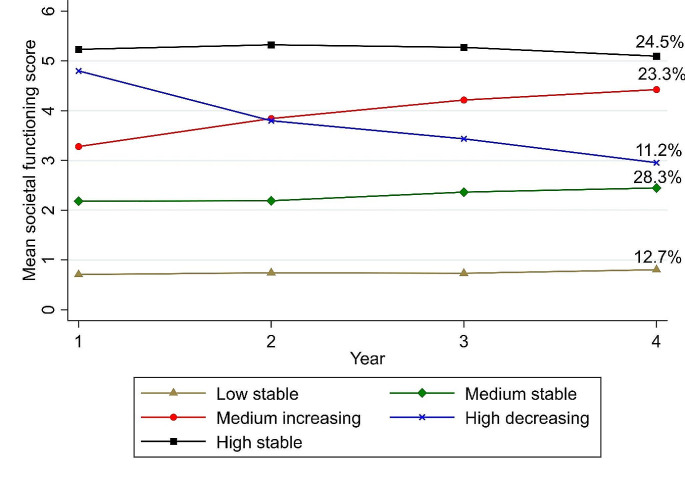
Societal recovery scores subdivided in the five classes identified in the LCGA over a four-year trajectory. Patients scoring a five or six are considered to be in societal recovery

**Fig. 2:**
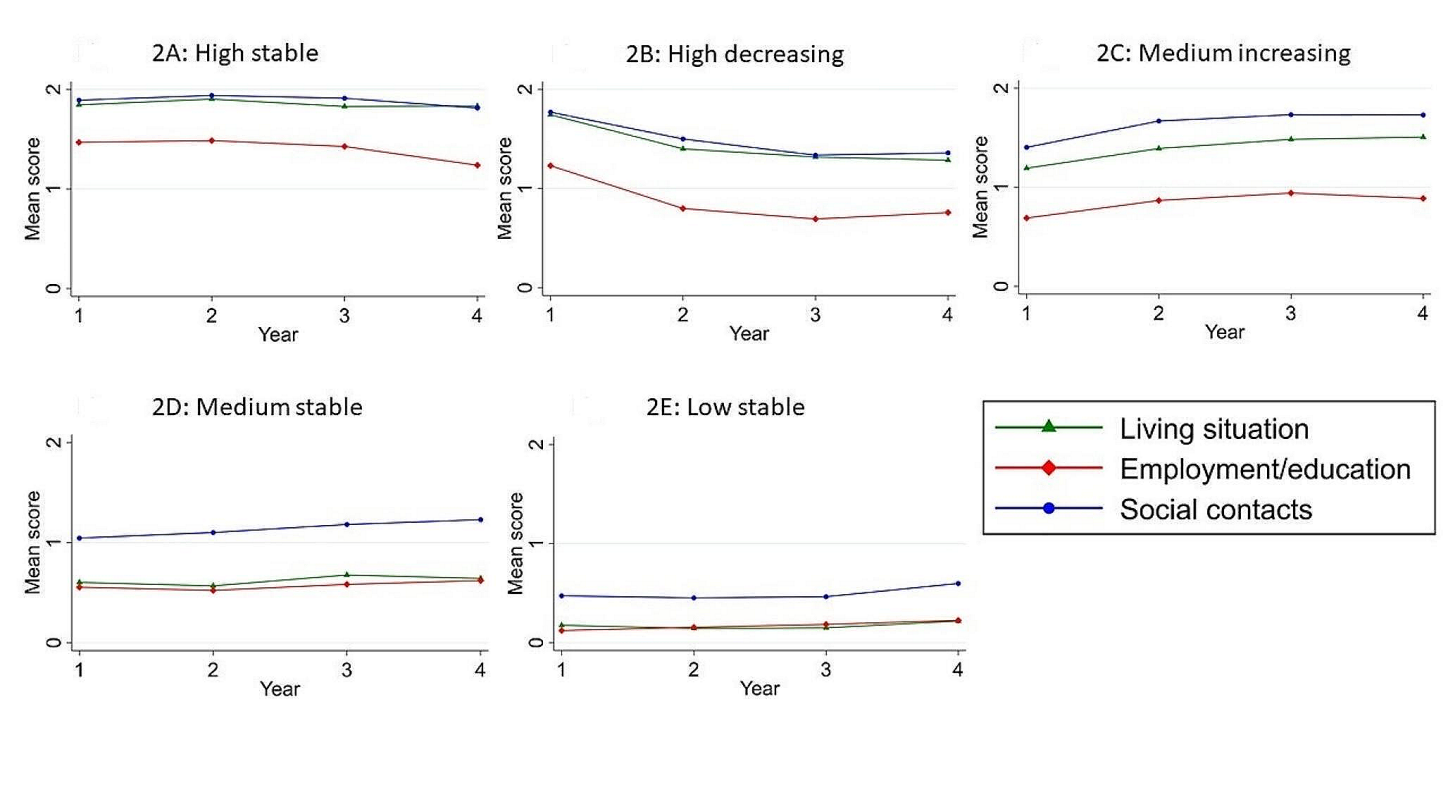
Scores on individual items of the functional recovery tool (living situation, employment/education and social contacts) subdivided in the five classes identified in the LCGA over a four-year trajectory. A score of two indicates independence, one indicates partial independence and zero indicates no independence

Six latent class growth models were made, ranging from one to six latent classes in order to identify the optimal number of latent classes. Table 2 shows the fit indices for the models with the increasing number of classes. For all the models a global solution was found as indicated by the replication of the likelihood ratio. Based on the VLMRT and the BIC, the model with five classes fitted the data best, however the p-value for the BLRT for the six classes solution indicated a better fit than a five classes solution. Based on the lowest BIC, a higher entropy and the VLRMT, the five classes solution was selected as the most optimal solution. Figure 1 shows the trajectories of the societal recovery score for the five identified classes over a period of four years. Three subgroups displayed a stable trajectory, a high stable subgroup (24.5%), a medium stable subgroup (28.3%) and a low stable subgroup (12.7%), accounting for a total of 65.5% of the participants. A fourth subgroup started high and declined to a medium level (11.2%) in the observed four years and the final subgroup started at a medium level and increased to a high level (23.3%). Figure 2 shows the trajectories for the three individual items of the functional recovery tool (daily living, employment/education and social contacts) subdivided by the five subgroups. For all five groups the employment/education item was consistently scoring the lowest, while the social contacts item scored the highest. For the low stable and the medium stable subgroups, the item for living situation scores as low as the employment/education item. For the other three subgroups with more favourable societal recovery trajectories the living situation item scored similar to the social contact item. The trends of the three individual items were similar to the trend of sum score of the FRt for all five subgroups.

**Table 2:** LCGA fit indices for increasing class solutions LCGA

Number of classes	Free parameters	BIC	Entropy	Proportion of the sample per class	VLMRT		BLRT	
				range	2LL	p-value	2LL	p-value
1	6	16468.948						
2	9	14637.223	0.831	0.44–0.56	1853.617	**< 0.0001**	1772.642	**< 0.0001**
3	12	14263.280	0.767	0.18–0.43	395.834	**0.0055**	395.834	**< 0.0001**
4	15	14074.419	0.746	0.13–0.35	210.75	**< 0.0001**	210.752	**< 0.0001**
5	18	14048.497	0.699	0.11–0.28	47.814	**0.0019**	45.725	**0.0024**
6	21	14054.633	0.657	0.09–0.25	15.755	0.1319	15.755	**< 0.0001**

Note: *P*-values < 0.05 are in boldface. 2LL = 2 time the loglikelihood difference; BIC = Bayesian Information Criteria; BLRT = Bootstrapped Likelihood Ratio Test; VLMRT = Vuong-Lo-Mendell-Rubin

### Between class differences

In a secondary analysis we compared baseline characteristics of classes identified in the LCGA with a similar baseline societal recovery score with distinct trajectories. Specifically, we compared the high stable subgroup with the high decreasing subgroup and the medium stable with the medium increasing subgroup. In appendix 1 the results of the univariate analysis are shown. Based on the univariate analysis thirteen predictors were used in the first model comparing the high stable subgroup with the high decreasing subgroup (sex, symptomatic recovery, happiness, nicotine use, cannabis use, somatic disease, hospitalisation in the last year, physically active, education, PANSS positive, PANSS negative, HoNOS behaviour, HoNOS limitations). Fourteen predictors were used in the first model comparing the medium stable subgroup with the medium increasing subgroup (symptomatic recovery, alcohol use, hospitalisation in the last year, physically active, education, type of antipsychotic, time since onset, age at onset, PANSS positive, PANSS negative, PANSS generic, HoNOS behaviour, HoNOS limitations and total antipsychotic dose). Table 3 shows the results from the two logistic regression models after backward selection. Predictors for not deteriorating from a high level of societal recovery include a happiness score of seven or higher, a recent hospitalisation, being physically active, middle or higher education and fewer negative symptoms at baseline. Predictors for improving from a medium level of societal recovery to a high level of recovery include fewer positive and negative symptoms, fewer behavioural problems and fewer physical and cognitive impairments at baseline.

**Table 3:** Predictors for class membership comparing the high stable group to the high decreasing group and the medium stable group to the medium increasing group

High stable (0) versus high decreasing (1) (*n* = 526)					Medium stable (0) versus medium increasing (1) (*n* = 762)			
Predictor	Odds ratio	95%CI	P-value		Predictor	Odds ratio	95%CI	P-value
Happiness ^a^	0.602	0.393; 0.921	0.029		PANSS positive	0.796	0.669; 0.947	0.010
Hospitalisation	0.415	0.194; 0.888	0.023		PANSS negative	0.785	0.666; 0.926	0.004
Physical activity	0.585	0.394; 0.868	0.020		HoNOS behaviour	0.586	0.381; 0.903	0.016
Lower education ^b^	2.675	1.229; 5.821	0.027		HoNOS limitations	0.609	0.484; 0.768	>0.001
Middle education ^b^	1.831	0.841; 3.986	0.170		Constant	4.174	2.491; 6.994	>0.001
Higher education ^b^	1.109	0.508; 2.421	0.795					
PANSS negative	1.460	1.106; 1.928	0.003					
Constant	0.282	0.113; 0.703	0.001					

PANSS = The Positive and Negative Syndrome Scale; HoNOS = The Health of the Nation Outcome Scales.

^a^ dichotomised score where a score of seven or higher on a scale from 0 to 10 is considered “happy”.

^b^ Missing education is used as the reference value.

## Discussion

The present study investigated trajectories of societal recovery in people who were diagnosed with a psychotic disorder at least five years prior to the first assessment. Specifically, patient subgroups characterized by homogeneous trajectories of societal recovery were identified. Approximately two-thirds showed a stable societal functioning trajectory over four years. A relatively small portion functions at a low level and does not show improvements in the observed four years. There are more people improving from a medium level to a high level than people worsening from a high level to a medium level. Predictors for an improvement in societal recovery are fewer positive and negative symptoms, fewer behavioural problems and fewer physical and cognitive impairments. Predictors for worsening from a high level are lower happiness, no hospitalisation in the past year, low physical activity, lower education and more negative symptoms.

Previous research investigating societal recovery trajectories in people with a psychotic disorder focused on recovery after a FEP [15, 16, 40], while in the present study only people in long term care were included. As our sample is characterized by longer illness duration, we expected to find lower societal functioning in comparison to these FEP samples [41]. A recent study on individuals enrolled in early intervention services confirmed this expectation as it reported a relatively small class (9.4%) that started low and improved to a moderate level of functioning during the observation period of one year [16]. In contrast, in the samples of the two other FEP studies [15, 40] the majority of participants started low and did not show improvement during the study period. We categorized only 12.7% of our sample in the low stable class, that said the medium stable class still showed fairly poor societal functioning, without improvement over time. Together these two classes represent about 40% of the participants. This difference between these studies and the current study in levels of functioning could be due to differences in population characteristics (e.g., younger, more recent onset and more homogeneous diagnoses), treatment, or the operationalization of social and occupational recovery. Of note is that while the first year after a FEP is often considered as a critical period for recovery [11–13], our findings indicate that the number of patients that still improve more than five years post-FEP are higher than those that deteriorate. This confirms that even in a population in care for almost 20 years on average, societal recovery can still be achieved. Important to note however that this was only achieved by people who already had a medium level of societal functioning at baseline.

A number of predictors of improvement and decline were investigated. Severity of psychosis symptoms was found to be a key predictor. Negative symptoms was the only predictor that predicted deteriorating from a high level of societal functioning as well as not improving societal functioning from a medium level. Secondly, a low level of positive symptoms was predictive of achieving societal recovery. This is in line with previous research that classified patients in four states of recovery based on symptomatic, societal and personal recovery [8]. In this study individuals in a recovery state characterized by a relatively high level of positive symptoms and low negative symptoms displayed a higher level of societal functioning than individuals in a state characterized by low positive and high negative symptoms. In the current study psychotic symptoms and behavioural problems were predictive for not reaching societal recovery for those that started at a medium level of societal recovery. Although we cannot be sure that this relationship is causal, it does show that more symptoms and behavioural problems are associated with lower societal functioning.

Notably, hospitalization in the previous year was a predictor for maintaining a high level of recovery. This might seem counterintuitive [42], but could be an indication that timely hospitalisation when a patient shows symptomatic decline can prevent societal recovery loss. For some individualists, not being hospitalised when it would be prudent could indicate withdrawal from care. A cross-sectional study found a similar counterintuitive result in patients after a FEP. In this study, psychiatric hospitalisations were predictive for clinical and societal recovery [41]. Their explanation was that early hospitalisations in FEP was indicative for a higher intensity of care in the initial phase of illness. Alternatively, this finding could be an indication of resilience. Although hospitalisation aims to promote recovery, the psychotic relapse itself likely diminished their societal functioning. Those who reported high societal functioning at baseline despite a psychotic relapse in the previous year severe enough to warrant a hospitalisation in the previous year, have shown to be resilient. This resilience could have helped them to maintain high societal functioning in the following years.

In this study, the employment component of societal functioning consistently lacked behind. Even the individuals in the three subgroups that exhibited societal recovery at some point in their trajectory, often did not reach full independence in this area. This is in line with previous research that showed low employment rates in individuals with a psychotic disorder [7, 43]. This might be explained by the fact that for many, a FEP develops during a crucial phase of gaining early work experience and obtaining education. Moreover, stigma can further hinder re-entering the workforce [1, 7]. These disadvantages might carry through to the later stages of life as exemplified by our finding that lower levels of education at baseline predicted decline of societal functioning at follow-up. Employment rate for people remain low despite increased focus in research and practice [1, 44]. A recent scoping review concluded that while interventions such as Individual Placement and Support (IPS) and Early Intervention (EI) can be successful at obtaining employment more focus is needed to keep employment [44]. Especially IPS focusses on finding employment fast and moved away from educational activities, our results show that lower education might still be a barrier.

These results and previous results [8] show that people in long term care are not a homogenous group when it comes to societal, personal and symptomatic recovery. While for some individuals these different forms of recovery go hand in hand, for many they do not [8, 45, 46]. This supports a personalised approach in which treatment goals should be assessed and prioritised in dialogue with the patient. Some might benefit from a shift of treatment focus from symptomatic recovery to societal and personal recovery. To identify those at risk of deteriorating, future research could focus on developing prediction models to predict changes in societal recovery. A recent review showed that the clinical impact of clinical psychiatry prediction models is yet to be tested in practice [47]. Nonetheless, the potential of such models is evident and have shown equally high accuracy as models used for cancer and cardiovascular disease [47].

An important strength of this study is that we analysed data from almost 1500 people with a psychotic disorder. This far exceed the advised minimum number of 500 to accurately identify the correct LCGA model and number of classes [48]. Moreover, due to the high number of patients and the large amount of information available in the PHAMOUS dataset we were able to include a diverse set of potential predictors. Additionally, we adressed a gap in literature since this study focussed on individuals in long term care, while most studies have characterised societal recovery trajectories in FEP. When interpreting these results, it is essential to account for the limitations of this study. Firstly, the FRt is a relatively brief and basal assessment of three aspects of functioning. However psychometric properties of the FRt have been shown to be adequate to good and the FRt has been shown to be sensitive to change [24, 25]. Secondly, because the PHAMOUS database is a naturalistic dynamic cohort database, loss to follow-up due to full recovery or relapse is a potential source of selection bias. Patients who were diagnosed at least five years ago were included when they had at least two measurements in four years. This might have resulted in an underestimation of the size of the low recovery group, who might have missed screenings more frequently. Similarly, the size of the high recovery group could have been underestimated because of loss to follow-up due to getting out of care. Thirdly, most patients included in the PHAMOUS study are receiving care from health care providers in the northern part of the Netherlands. This is a relatively rural area with only a small portion of patients with a non-white ethnicity, which is reflected in our sample, limiting the generalisability of the results. Lastly, the follow-up may not have been long enough to identify fluctuations in recovery. For example, the group that showed improvement from a medium level and the group that showed decline from a high level might not be distinct groups, but part of a singular group that fluctuates between a medium and high level of recovery over a longer period than four years.

## Conclusion

This study showed that trajectories of societal recovery of people in long term care for a psychotic disorder are heterogeneous. The majority showed a stable trajectory over four years and there were more individuals achieving societal recovery than individuals declining. This underlines that societal recovery is possible for people in long term care. Predictors for improvement to a high level of societal functioning are mainly related to symptoms and behavioural problems, while predictors for a decline in functioning from a high level are related to physical activity, happiness and level of education.

## Electronic supplementary material

Below is the link to the electronic supplementary material.

Supplementary Material 1

## Data Availability

Under the General Data Protection Regulation, our data is considered pseudonymised rather than anonymised, and is therefore still regarded as personal data. Given that participants have not given informed consent to have their personal data publicly shared, we are legally and ethically not allowed to publicly post our data-set. Data is therefore only available upon request to the Data Science Center of the Rob Giel Research Center (email: e.visser03@ umcg.nl).

## References

[1] Rinaldi M, Killackey E, Smith J et al (2010) First episode psychosis and employment: a review. Int Rev Psychiatry 22:148–162. 10.3109/09540261003661825 20504055 10.3109/09540261003661825

[2] Harvey PD, Bosia M, Cavallaro R et al (2022) Cognitive dysfunction in schizophrenia: an expert group paper on the current state of the art. Schizophr Res Cogn 29. 10.1016/j.scog.2022.100249 10.1016/j.scog.2022.100249 PMC8956816 35345598

[3] van Aken BC, Bakia A, Wierdsma AI et al (2021) UP’S: a Cohort Study on Recovery in psychotic disorder patients: design protocol. Front Psychiatry 11:1–13. 10.3389/fpsyt.2020.609530 10.3389/fpsyt.2020.609530 PMC7874019 33584375

[4] Rosenheck R, Stroup S, Keefe RSE et al (2005) Measuring outcome priorities and preferences in people with schizophrenia. Br J Psychiatry 187:529–534. 10.1192/bjp.187.6.529 16319405 10.1192/bjp.187.6.529

[5] Fifer S, Keen B, Newton R et al (2022) Understanding the Treatment Preferences of People Living with Schizophrenia in Australia; a patient Value Mapping Study. Patient Prefer Adherence 16:1687–1701. 10.2147/PPA.S366522 35898923 10.2147/PPA.S366522 PMC9309312

[6] Tohen M, Khalsa HMK, Salvatore P et al (2016) The McLean-Harvard first-episode project: early course in 114 cases of first-episode nonaffective psychoses. J Clin Psychiatry 77:781–788. 10.4088/JCP.14m09414 27232651 10.4088/JCP.14m09414

[7] Ajnakina O, Stubbs B, Francis E et al (2021) Employment and relationship outcomes in first-episode psychosis: a systematic review and meta-analysis of longitudinal studies. Schizophr Res 231:122–133. 10.1016/j.schres.2021.03.013 33839370 10.1016/j.schres.2021.03.013

[8] Castelein S, Timmerman ME, Van Der Gaag M et al (2021) Clinical, societal and personal recovery in schizophrenia spectrum disorders across time: States and annual transitions. Br J Psychiatry 219:401–408. 10.1192/bjp.2021.48 35048855 10.1192/bjp.2021.48 PMC8529640

[9] Jobe TH, Harrow M (2005) Long-term outcome of patients with schizophrenia: a review. Can J Psychiatry 50:892–900. 10.1177/070674370505001403 16494258 10.1177/070674370505001403

[10] Morgan C, Lappin J, Heslin M et al (2014) Reappraising the long-term course and outcome of psychotic disorders: the AESOP-10 study. Psychol Med 44:2713–2726. 10.1017/S0033291714000282 25066181 10.1017/S0033291714000282 PMC4134320

[11] Álvarez-Jiménez M, Parker AG, Hetrick SE et al (2011) Preventing the second episode: a systematic review and meta-analysis of psychosocial and pharmacological trials in first-episode psychosis. Schizophr Bull 37:619–630. 10.1093/schbul/sbp129 19900962 10.1093/schbul/sbp129 PMC3080698

[12] Birchwood M, Todd P, Jackson C (1998) Early intervention in psychosis. The critical period hypothesis. Br J Psychiatry Suppl 172:53–59 9764127

[13] Crumlish N, Whitty P, Clarke M et al (2009) Beyond the critical period: longitudinal study of 8-year outcome in first-episode non-affective psychosis. Br J Psychiatry 194:18–24. 10.1192/bjp.bp.107.048942 19118320 10.1192/bjp.bp.107.048942

[14] Santesteban-Echarri O, Paino M, Rice S et al (2017) Predictors of functional recovery in first-episode psychosis: a systematic review and meta-analysis of longitudinal studies. Clin Psychol Rev 58:59–75. 10.1016/j.cpr.2017.09.007 29042139 10.1016/j.cpr.2017.09.007

[15] Hodgekins J, Birchwood M, Christopher R et al (2015) Investigating trajectories of social recovery in individuals with first-episode psychosis: a latent class growth analysis. Br J Psychiatry 207:536–543. 10.1192/bjp.bp.114.153486 26294371 10.1192/bjp.bp.114.153486 PMC4664858

[16] van der Ven E, Scodes J, Basaraba C et al (2020) Trajectories of occupational and social functioning in people with recent-onset non-affective psychosis enrolled in specialized early intervention services across New York state. Schizophr Res 222:218–226. 10.1016/j.schres.2020.05.051 32513547 10.1016/j.schres.2020.05.051 PMC8273912

[17] Hall M-H, Holton KM, Öngür D et al (2019) Longitudinal trajectory of early functional recovery in patients with first episode psychosis. Schizophr Res 209:234–244. 10.1016/j.schres.2019.02.003 30826261 10.1016/j.schres.2019.02.003 PMC7003957

[18] Abdin E, Chong SA, Vaingankar JA et al (2017) Trajectories of positive, negative and general psychopathology symptoms in first episode psychosis and their relationship with functioning over a 2-year follow-up period. PLoS ONE 12:1–16. 10.1371/journal.pone.0187141 10.1371/journal.pone.0187141 PMC5667842 29095875

[19] Jääskeläinen E, Juola P, Hirvonen N et al (2013) A systematic review and meta-analysis of recovery in schizophrenia. Schizophr Bull 39:1296–1306. 10.1093/schbul/sbs130 23172003 10.1093/schbul/sbs130 PMC3796077

[20] Xia J, Merinder LB, Belgamwar MR (2011) Psychoeducation for schizophrenia. Schizophr Bull 37:21–22. 10.1093/schbul/sbq138 21147896 10.1093/schbul/sbq138 PMC3004189

[21] Bartels-Velthuis AA, Visser E, Arends J et al (2018) Towards a comprehensive routine outcome monitoring program for people with psychotic disorders: the Pharmacotherapy Monitoring and Outcome Survey (PHAMOUS). Schizophr Res 197:281–287. 10.1016/j.schres.2018.01.016 29395613 10.1016/j.schres.2018.01.016

[22] American Psychiatric Association DSM-5 Task Force (2013) Diagnostic and statistical manual of mental disorders: DSM-5. American Psychiatric Association, Arlington, VA

[23] American Psychiatric Association Task Force on DSM-IV (1994) Diagnostic and statistical manual of mental disorders: DSM-IV. American Psychiatric Association, Washington, DC

[24] Swildens WE, Visser E, Bähler M et al (2018) Functional recovery of individuals with serious mental illnesses: development and testing of a new short instrument for routine outcome monitoring. Psychiatr Rehabil J 41:341–350. 10.1037/prj0000320 30507243 10.1037/prj0000320

[25] Wiersma D, Visser E, Bahler M et al (2015) Functionele remissie bij mensen met een ernstige psychiatrische aandoening; psychometrische eigenschappen van een nieuw ROM-instrument. Tijdschr Psychiatr 57:395–404 26073833

[26] Roosenschoon BJ, Kamperman AM, Deen ML et al (2019) Determinants of clinical, functional and personal recovery for people with schizophrenia and other severe mental illnesses: a cross-sectional analysis. PLoS ONE 14:1–14. 10.1371/journal.pone.0222378 10.1371/journal.pone.0222378 PMC6750648 31532805

[27] Chang WC, Kwong VWY, Chan GHK et al (2016) Prediction of functional remission in first-episode psychosis: 12-month follow-up of the randomized-controlled trial on extended early intervention in Hong Kong. Schizophr Res 173:79–83. 10.1016/j.schres.2016.03.016 27017490 10.1016/j.schres.2016.03.016

[28] Chan SKW, So HC, Hui CLM et al (2015) 10-Year Outcome Study of an early intervention program for psychosis compared with Standard Care Service. Psychol Med 45:1181–1193. 10.1017/S0033291714002220 25233868 10.1017/S0033291714002220

[29] Ventura J, Subotnik KL, Guzik LH et al (2011) Remission and recovery during the first outpatient year of the early course of schizophrenia. Schizophr Res 132:18–23. 10.1016/j.schres.2011.06.025 21764563 10.1016/j.schres.2011.06.025 PMC3172347

[30] Moriarty A, Jolley S, Callanan MM, Garety P (2012) Understanding reduced activity in psychosis: the roles of stigma and illness appraisals. Soc Psychiatry Psychiatr Epidemiol 47:1685–1693. 10.1007/s00127-012-0475-z 22366910 10.1007/s00127-012-0475-z

[31] Kay SR, Fiszbein A, Opler LA (1987) The positive and negative syndrome scale (PANSS) for schizophrenia. Schizophr Bull 13:261–276. 10.1093/schbul/13.2.261 3616518 10.1093/schbul/13.2.261

[32] Wing JK, Beevor AS, Curtis RH et al (1998) Health of the Nation Outcome scales (HoNOS): Research and development. Br J Psychiatry 172:11–18. 10.1192/bjp.172.1.11 9534825 10.1192/bjp.172.1.11

[33] Abdel-Khalek AM (2006) Measuring happiness with a single-item scale. Soc Behav Personal Int J 34:139–150. 10.2224/sbp.2006.34.2.139

[34] Lako IM, Van Den Heuvel ER, Knegtering H et al (2013) Estimating dopamine d2 receptor occupancy for doses of 8 antipsychotics: a meta-analysis. J Clin Psychopharmacol 33:675–681. 10.1097/JCP.0b013e3182983ffa 23948784 10.1097/JCP.0b013e3182983ffa

[35] Jung T, Wickrama KAS (2008) An introduction to latent class growth analysis and growth mixture modeling. Soc Personal Psychol Compass 2:302–317. 10.1111/j.1751-9004.2007.00054.x

[36] Muthén L, Muthén B (2007) Mplus user’s guide (version 7). Los Angeles, Muthén & Muthén

[37] Lo Y, Mendell NR, Rubin DB (2001) Testing the number of components in a normal mixture. Biometrika 88:767–778

[38] Nylund KL, Asparouhov T, Muthén BO (2007) Deciding on the number of classes in latent class analysis and growth mixture modeling: a Monte Carlo simulation study. Struct Equ Model 14:535–569. 10.1080/10705510701575396

[39] Andreasen NC, Carpenter WTJ, Kane JM et al (2005) Remission in schizophrenia: proposed criteria and rationale for consensus. Am J Psychiatry 162:441–449. 10.1176/appi.ajp.162.3.441 15741458 10.1176/appi.ajp.162.3.441

[40] Chang WC, Chu AOK, Kwong VWY et al (2018) Patterns and predictors of trajectories for social and occupational functioning in patients presenting with first-episode non-affective psychosis: a three-year follow-up study. Schizophr Res 197:131–137. 10.1016/j.schres.2018.01.021 29395604 10.1016/j.schres.2018.01.021

[41] Altamura AC, Serati M, Buoli M (2015) Is duration of illness really influencing outcome in major psychoses? Nord J Psychiatry 69:1685–1699. 10.3109/08039488.2014.990919 10.3109/08039488.2014.990919 25768662

[42] Harvey PD, Loewenstein DA, Czaja SJ (2013) Hospitalization and psychosis: influences on the course of cognition and everyday functioning in people with schizophrenia. Neurobiol Dis 53:18–25. 10.1016/j.nbd.2012.10.022 23123218 10.1016/j.nbd.2012.10.022 PMC3574628

[43] Iyer S, Mustafa S, Gariépy G et al (2018) A NEET distinction: youths not in employment, education or training follow different pathways to illness and care in psychosis. Soc Psychiatry Psychiatr Epidemiol 53:1401–1411. 10.1007/s00127-018-1565-3 30094632 10.1007/s00127-018-1565-3 PMC6267132

[44] Aguey-Zinsou M, Scanlan JN, Cusick A (2023) A scoping and systematic review of employment processes and outcomes for young adults experiencing psychosis. Community Ment Health J 59:728–755. 10.1007/s10597-022-01056-z 36463531 10.1007/s10597-022-01056-z

[45] Frawley E, Cowman M, Lepage M, Donohoe G (2021) Social and occupational recovery in early psychosis: a systematic review and meta-analysis of psychosocial interventions. Psychol Med. 10.1017/S003329172100341X 34474696 10.1017/S003329172100341X PMC10106304

[46] Van Eck RM, Burger TJ, Vellinga A et al (2018) The relationship between clinical and personal recovery in patients with Schizophrenia Spectrum disorders: a systematic review and Meta-analysis. Schizophr Bull 44:631–642. 10.1093/schbul/sbx088 29036720 10.1093/schbul/sbx088 PMC5890469

[47] Meehan AJ, Lewis SJ, Fazel S et al (2022) Clinical prediction models in psychiatry: a systematic review of two decades of progress and challenges. Mol Psychiatry 27:2700–2708. 10.1038/s41380-022-01528-4 35365801 10.1038/s41380-022-01528-4 PMC9156409

[48] Sinha P, Calfee CS, Delucchi KL (2021) Practitioner’s guide to latent class analysis: methodological considerations and common pitfalls. Crit Care Med 49:e63–e79. 10.1097/CCM.0000000000004710 33165028 10.1097/CCM.0000000000004710 PMC7746621

