# Supplementary material for: Societal recovery trajectories in people with a psychotic disorder in long term care: a latent class growth analysis
Source: Soc Psychiatry Psychiatr Epidemiol. 2024 Jul 30;60(2):387–97. doi: 10.1007/s00127-024-02715-0 (PMC11839786; doi:10.1007/s00127-024-02715-0)
Supplement: Supplementary file 1 — Supplementary Material 1 [file 127_2024_2715_MOESM1_ESM.pdf]

Societal recovery trajectories in people with a psychotic disorder with a long duration of illness: a latent class growth analysis

Stijn Crutzen[1]*Ψ, Simone R Burger[2,3]Ψ, Ellen Visser[4], Helga K. Ising[5], PHAMOUS investigators** Mark van der Gaag[2,3], Stynke Castelein[1,7]

*Corresponding author: s.crutzen@lentis.nl

Ψ Shared first authors

** Johan Arends[6], Frederike Jörg[4], Gerdina Hendrika Maria Pijnenborg[6,7], Wim Veling[8]

1 Lentis Psychiatric Institute, Lentis Research, Hereweg 80, 9725 AG, Groningen, The Netherlands

2 Department of Clinical Psychology, VU University and Amsterdam Public Health Research Institute, Amsterdam, the Netherlands

3 Department of Psychosis Research and Innovation, Parnassia Psychiatric Institute, The Hague, the Netherlands

4 University of Groningen, University Medical Center Groningen, University Center Psychiatry,

Rob Giel Research Center, Hanzeplein 1 (CC72), 9713 GZ, Groningen, The Netherlands

5 Rivierduinen Institute for Mental Health Care, Sandifortdreef 19, 2333 ZZ Leiden, the Netherlands

6 GGZ Drenthe Mental Health Institution, Department of Psychotic Disorders, Dennenweg 9, 9404 LA,

Assen, The Netherlands

7 University of Groningen, Faculty of Behavioural and Social Sciences, Grote Kruisstraat 2/1, 9712 TS,

Groningen, The Netherlands

8 University of Groningen, University Medical Center Groningen, University Center for Psychiatry,

Psychosis Department, Hanzeplein 1 (CC60), 9713 GZ, Groningen, The Netherlands.

Appendix 1

Table 1. Univariate analysis comparing the high stable subgroup with the high decreasing subgroup for the categorical variables

Potential predictor	High stable	High decreasing	p-value
**Sex male, %**	**54.0%**	**66.7%**	**0.006**
Ethnicity Caucasian, %	91.4%	93.9%	0.316
**Symptomatic remission, %**	**73.0%**	**58.9%**	**0.004**
**Personal remission, %**	**69.4%**	**58.7%**	**0.020**
Alcohol use, %	53.3%	49.4%	0.407
**Nicotine use, %**	**36.3%**	**50.8%**	**0.006**
**Cannabis use, %**	**7.7%**	**12.8%**	**0.062**
**Somatic disease, %**	**39.6%**	**49.3%**	**0.035**
**Hospitalisation in the last year, %**	**12.3%**	**6.3%**	**0.039**
**Physically active >30min 4xw, %**	**54.3%**	**42.7%**	**0.014**
Main psychotic disorder diagnosis, %			0.601
Schizophrenia	41.8%	43.6%	
Delusional disorder	2.5%	4.2%	
Schizophreniform disorder	5.0%	6.1%	
Substance abuse psychosis	11.4%	7.3%	
Psychosis NOS	1.9%	3.0%	
Schizoaffective disorder	18.0%	15.2%	
Uknown psychotic disorder	19.4%	20.6%	
**Education, %**			
**Lower education**	**27.5%**	**39.6%**	**0.008**
**Middle education**	**31.8%**	**32.5%**	
**Higher education**	**40.7%**	**27.9%**	
Type of antipsychotic, %			0.113
Strong dopamine antagonist	39.2%	37.3%	
Partial dopamine antagonist	17.2%	11.8%	
Weak dopamine antagonist	24.8%	28.6%	
Other	12.9%	19.3%	
No antipsychotic	5.9%	3.1%	

P-values ≤0.1 are included in the first logistic regression model in the backward selection method

Table 2. Univariate analysis comparing the medium stable subgroup with the medium increasing subgroup for the categorical variables

**Potential predictor**	**Medium stable**	**Medium increasing**	**p-value**
Sex male, %	72.3%	68.6%	0.272
Ethnicity Caucasian, %	84.7%	86.3%	0.521
**Symptomatic remission, %**	**35.6%**	**49.8%**	**<0.001***
Personal remission, %	57.0	59.3	0.553
**Alcohol use, %**	**35.0%**	**45.5%**	**0.004***
Nicotine use, %	50.7%	47.7%	0.488
Cannabis use, %	11.8%	11.9%	0.995
Somatic disease, %	50.4%	48.4%	0.588
**Hospitalisation in the last year, %**	**16.2%**	**10.6%**	**0.028***
**Physically active >30min 4xw, %**	**34.8%**	**42.8%**	**0.026***
Main psychotic disorder diagnosis, %			0.204
Schizophrenia	56.5%	47.6%	
Delusional disorder	0.5%	0.9%	
Schizophreniform disorder	4.8%	6.7%	
Substance abuse psychosis	6.2%	7.9%	
Psychosis NOS	2.6%	3.2%	
Schizoaffective disorder	9.3%	13.7%	
Uknown psychotic disorder	20.1%	20.1%	
**Education, %**			**0.001***
**Lower education**	**58.9%**	**44.4%**	
**Middle education**	**20.7%**	**28.1%**	
**Higher education**	**20.4%**	**27.5%**	
**Type of antipsychotic, %**			**0.023***
**Strong dopamine antagonist**	**37.5%**	**33.4%**	
**Partial dopamine antagonist**	**11.1%**	**15.8%**	
**Weak dopamine antagonist**	**31.1%**	**24.2%**	
**Other**	**15.8%**	**22.4%**	
**No antipsychotic**	**4.4%**	**4.2%**	

P-values ≤0. 1 are included in the first logistic regression model in the backward selection method

Table 3. Univariate analysis comparing the high stable subgroup with the high decreasing subgroup and the medium stable subgroup with the medium increasing subgroup for the continues variables

Potential predictor	High stable	High decreasing	P-value
Age (mean), years	42.6	43.7	0.260
Time since onset (median), years	15	17	0.153
Age at onset (mean), years	26.0	25.9	0.891
**PANSS positive (median)**	**1.33**	**1.67**	**0.002**
**PANSS negative (median)**	**1.33**	**1.67**	**0.003**
PANSS generic (median)	1.00	1.00	0.113
Single item happiness (mean), 0-10	6.74	6.62	0.447
**HONOS behaviour (median)**	**0.00**	**0.00**	**0.002**
**HONOS limitations (median)**	**0.50**	**0.50**	**0.027**
Total antipsychotic Haloperidol equivalent dose (median), mg	5.00	5.00	0.137
**Potential predictor**	**Medium stable**	**Medium increasing**	**P-value**
Age (mean), years	44.9	43.7	0.298
**Time since onset (median), years**	**19**	**17**	**0.003**
**Age at onset (mean), years**	**24.5**	**25.9**	**0.036**
**PANSS positive (median)**	**2.00**	**1.67**	**<0.001**
**PANSS negative (median)**	**2.33**	**2.00**	**<0.001**
**PANSS generic (median)**	**1.50**	**1.00**	**0.001**
Single item happiness (mean), 0-10	6.48	6.53	0.701
**HoNOS behaviour (median)**	**0.33**	**0.00**	**0.002**
**HoNOS limitations (median)**	**1.00**	**0.50**	**<0.001**
**Total antipsychotic Haloperidol equivalent dose (median), mg**	**6.67**	**6.25**	**0.004**

P-values ≤0.1 are included in the first logistic regression model in the backward selection method. An independent samples t-test was used for normally distributed predictors (mean reported) and a Mann-Whitney test was used for non-normally distributed predicators (median reported). PANSS = The Positive and Negative Syndrome Scale; HoNOS = The Health of the Nation Outcome
